# Postprocessing of molecular docking poses using binding free energy calculations

**DOI:** 10.1186/1758-2946-4-S1-P42

**Published:** 2012-05-01

**Authors:** Kanin Wichapong, Wolfgang Sippl

**Affiliations:** 1 Department of Pharmaceutical Chemistry, Martin-Luther University Halle-Wittenberg, 06120, Halle(Saale), Germany

## 

The main key to success in structure-based drug discovery is the accurate prediction of binding affinities of hit compounds. Molecular docking and scoring functions are often used for this purpose. However, it is often found that the top-ranked docking poses do not represent the right binding mode, and frequently there is no correlation between docking score and biological data. Therefore, “post-processing” of docking poses has recently got attraction. In previous work [1], we have successfully computed binding free energies (MM/PBSA) of 222 Wee1 kinase inhibitors and used the derived validated models for virtual screening. In the current work, we extended our studies to a data set of PIM1 kinase inhibitors. Cross-docking studies showed that the correct binding mode of the inhibitors can be determined after applying a post-processing procedure. The top-ranked docking poses gave wrong binding mode (high RMSD values ~4.0 Å), whereas the top-ranked poses selected after postprocessing yielded RMSD values around 0.5 Å. Subsequently, the docking poses giving the lowest binding free energy were selected and these values were used to establish a correlation with experimental data. A significant correlation between ΔG_cal._ and ΔG_exp_ (*r^2^* = 0.58) was obtained. To summarize, the protocol described in the current work can be used for postprocessing of protein-ligand docking poses and for predicting biological activities of novel hits. Therefore, the protocol can be applied for structure-based optimization of hit molecules.
